# Multiple Sclerosis in the Campania Region (South Italy): Algorithm Validation and 2015–2017 Prevalence

**DOI:** 10.3390/ijerph17103388

**Published:** 2020-05-13

**Authors:** Marcello Moccia, Vincenzo Brescia Morra, Roberta Lanzillo, Ilaria Loperto, Roberta Giordana, Maria Grazia Fumo, Martina Petruzzo, Nicola Capasso, Maria Triassi, Maria Pia Sormani, Raffaele Palladino

**Affiliations:** 1 Multiple Sclerosis Clinical Care and Research Centre, Department of Neuroscience, Reproductive Science and Odontostomatology, Federico II University, Via Sergio Pansini 5, 80131 Naples, Italy; vincenzo.bresciamorra2@unina.it (V.B.M.); robertalanzillo@libero.it (R.L.); martinapetruzzo@gmail.com (M.P.); nicolacapasso91@gmail.com (N.C.); 2 Department of Public Health, Federico II University, 80131 Naples, Italy; ilaria.loperto@gmail.com (I.L.); triassi@unina.it (M.T.); raffaele.palladino@unina.it (R.P.); 3 Campania Region Healthcare System Commissioner Office, 80131 Naples, Italy; r.giordana@soresa.it; 4 Regional Healthcare Society (So.Re.Sa), 80131 Naples, Italy; m.fumo@soresa.it; 5 Biostatistics Unit, Department of Health Sciences, University of Genoa, 16121 Genoa, Italy; mariapia.sormani@unige.it; 6 Department of Primary Care and Public Health, Imperial College, London SW7 2AZ, UK

**Keywords:** multiple sclerosis, prevalence, routinely collected healthcare data, Italy

## Abstract

We aim to validate a case-finding algorithm to detect individuals with multiple sclerosis (MS) using routinely collected healthcare data, and to assess the prevalence of MS in the Campania Region (South Italy). To identify individuals with MS living in the Campania Region, we employed an algorithm using different routinely collected healthcare administrative databases (hospital discharges, drug prescriptions, outpatient consultations with payment exemptions), from 1 January 2015 to 31 December 2017. The algorithm was validated towards the clinical registry from the largest regional MS centre (n = 1460). We used the direct method to standardise the prevalence rate and the capture-recapture method to estimate the proportion of undetected cases. The case-finding algorithm including individuals with at least one MS record during the study period captured 5362 MS patients (females = 64.4%; age = 44.6 ± 12.9 years), with 99.0% sensitivity (95% CI = 98.3%, 99.4%). Standardised prevalence rate per 100,000 people was 89.8 (95% CI = 87.4, 92.2) (111.8 for females [95% CI = 108.1, 115.6] and 66.2 for males [95% CI = 63.2, 69.2]). The number of expected MS cases was 2.7% higher than cases we detected. We developed a case-finding algorithm for MS using routinely collected healthcare data from the Campania Region, which was validated towards a clinical dataset, with high sensitivity and low proportion of undetected cases. Our prevalence estimates are in line with those reported by international studies conducted using similar methods. In the future, this cohort could be used for studies with high granularity of clinical, environmental, healthcare resource utilisation, and pharmacoeconomic variables.

## 1. Introduction

Multiple sclerosis (MS) is the leading cause of disability from central nervous system disease in young adults [1,2]. Prevalence of MS has increased by 10% from 1990, reaching 91–164 cases per 100,000 in North America, Western Europe and Australasia [1]. Clinical onset is generally in early adult life, though there is increased awareness of presentation in childhood [2]. Prevalence of MS is similar in preteen boys and girls, but then progressively increases throughout the lifetime among women, with a 2:1 sex ratio in favour of women in the sixth decade of life [1].

In the past decades, fifteen disease-modifying therapies (DMTs) for MS have been developed within randomised controlled trials (RCTs), directly comparing different DMTs (or placebo) in a short time frame (24–36 months), and for specific clinical outcome measures (e.g., relapses, disability) [3,4]. In the meanwhile, worldwide MS centres have developed MS registries to provide meaningful information on the natural history of MS, and on the long-term safety and effectiveness of DMTs in the real-life [5,6,7]. However, RCTs and MS registries do not include overall healthcare resource utilisation [6,8,9,10], and, not least, hold potential risks from patient selection (e.g., with earlier and more educated patients being more likely to visit MS centres and participate into research), and follow-up (e.g., with patients doing poorly being more likely to be lost to follow-up) [11,12].

Population-based studies can overcome these limitations. Datasets of routinely collected healthcare data, derived from both publicly-funded and private healthcare systems [13,14], provide standardised healthcare resource utilisation in the long-term (e.g., diagnoses, procedures, medications), and on fully representative populations [15]. As such, population-based studies can provide a detailed description of disease epidemiology, comorbidities and treatment pathways [15], also thanks to the linkage to clinical registries [16,17].

Hereby, we aim to: (1) validate an algorithm to identify MS cases using routinely collected healthcare administrative databases; (2) estimate the 2015–2017 prevalence of MS in the Campania Region (South Italy) and in its provinces; and (3) estimate the proportion of undetected cases.

## 2. Methods

### 2.1. Study Design and Setting

This is a population-based study, obtained from the retrospective analysis of routinely collected healthcare data of individuals residing in the Campania Region (South Italy) from 2015 to 2017 (population on 1 January 2018: 5,826,860 with 2,841,049 males and 2,985,811 females) (http://dati.istat.it/).

The Italian National Healthcare Service (NHS) operates under the principles of universal coverage and is organised at the national, regional, and local levels. The central government controls the distribution of tax revenues for publicly financed healthcare, whilst the regions are responsible to organise and deliver healthcare services through Local Health Authorities (Azienda Sanitaria Locale (ASL)) [18]. In the Campania Region, there are seven Local Health Authorities (ASL Avellino, Benevento, Caserta, Napoli 1 Centro, Napoli 2 Nord, Napoli 3 Sud, and Salerno), overall including 10 qualified MS centres, complying with regulatory indications for DMT prescription and management [8,19,20]. Healthcare services delivered out of the Campania Region (e.g., DMT prescriptions, hospital admissions, outpatient consultations) are then reported to the Campania Region for refund purposes.

The study was approved by the Federico II Ethics Committee (355/19). All patients signed informed consent authorising the use of anonymised, routinely collected healthcare data, in line with data protection regulation (GDPR EU2016/679). The study was performed in accordance with good clinical practice and the Declaration of Helsinki.

### 2.2. Population

The dataset was created by merging different data sources of the Campania Region. In particular, the cohort comprised all individuals alive at the prevalence day (1 January 2018) who had at least one record from the following databases [21]:

1. Hospital Discharge Record database, which included all admissions in the study period with an ICD-9 CM code of MS as one of the discharges diagnoses.
2. Regional Drug Prescription database, which included all MS-specific DMTs prescribed in the study period (e.g., Alemtuzumab, Dimethyl Fumarate, Fingolimod, Glatiramer Acetate, Interferon Beta-1a, Interferon Beta-1b, Natalizumab, Peg-Interferon Beta-1a, Teriflunomide).
3. Outpatient database, which included all outpatient consultations with an MS-specific exemption from co-payment records (as defined in the Healthcare Co-payment Database).

Hospital admissions, DMT prescriptions, and outpatient consultations delivered out of the Campania Region were reported to the Campania Region Healthcare Regulatory Society (So.Re.Sa.) for refund purposes and then included in the above-mentioned datasets.

From the database, individuals with a diagnosis of MS not resident in the Campania Region were filtered out. Patient unique identifier code was fully anonymised by the Campania Region Healthcare Regulatory Society (So.Re.Sa.) before releasing the datasets. As the same anonymisation algorithm was used across datasets, data merging was possible. As an additional measure of patient privacy protection, the only demographic information retained from each dataset was year of birth, sex, education attainment, and local health authority the individual was registered with. We also extracted type and frequency of DMT prescription, type and frequency of access to healthcare facilities, and type of exemption from co-payment records (e.g., MS-specific, disability due to other conditions, low household income).

**Figure 1:**
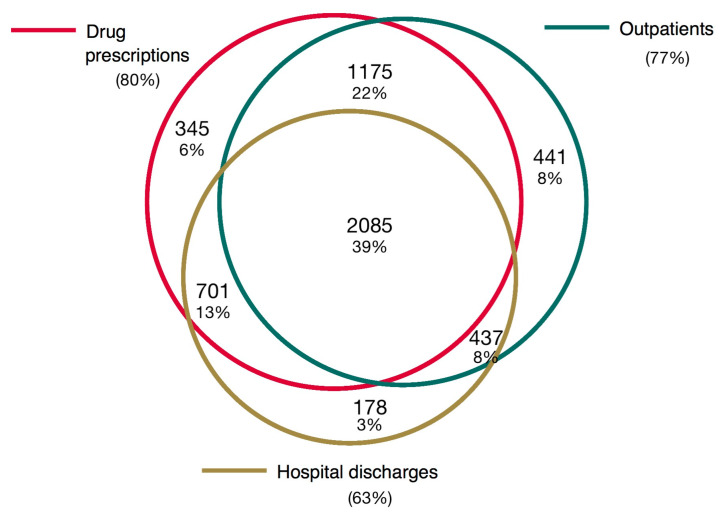
Venn diagram.

Proportions of patients identified from Hospital Discharge Record database, Regional Drug Prescription database, and Outpatient database are presented in Figure 1.

The Venn diagram shows the number and the proportions of patients identified from different data sources from the overall population (n = 5362).

### 2.3. Clinical Dataset

We extracted individuals with a diagnosis of MS and resident in the Campania Region from the clinical registry of the MS Clinical Care and Research Centre, at the “Federico II” University of Naples, Italy, from 2015 to 2017 (n = 1460). This cohort is part of the Italian MS Registry [7] and has already been used for a number of cohort studies [22,23]. Of note, a recent meta-analysis defined this cohort at moderate risk of bias, compared with the serious risk of other similar cohorts [24,25].

Anonymisation was performed using the same algorithm as for routinely collected healthcare data to allow data linkage.

### 2.4. Statistical Analysis

Missing data was present for year of birth (2.5%), and Local Health Authorities of registration (8.8%). A missing pattern analysis involving both graphical and statistical methods (e.g., logistic regression models) was carried out to deem data being missing at random. Thus, we used multiple imputation by chained equations (10 copies) to estimate missing data for age and local health authority. We included the following covariates in the imputation model: sex, number of records for each patient within the study period, DMT prescription (or no DMT prescription), hospital admissions (or no hospital admissions), outpatient consultations (or no outpatient consultations), and MS centre where healthcare was delivered.

Considering that previously published MS case-finding algorithms to identify people with MS using administrative healthcare databases varied in relation to the number of MS records considered for each patient for case identification [26,27,28,29], we aimed to validate two versions of the algorithm (aim 1), which considered the presence of: 1) at least one MS record during the study period; and 2) at least two MS records during the study period. To validate the algorithm, we merged our dataset with a clinical registry and identified individuals who accessed the MS Clinical Care and Research Centre at the “Federico II” University of Naples using the information provided by the algorithm. Then, we assessed the ability of the candidate algorithms to capture people with MS from the clinical registry using sensitivity, specificity, positive and negative predictive values, area under the curve (AUC), and κ-statistics as measures of agreement [30].

To capture MS prevalence (aim 2), age-standardised prevalence rates were calculated for the whole cohort and then stratified by sex using the direct standardisation method. The European population in 2018 was considered as a reference population. To assess differences in the prevalence ratios in the five provinces of the Campania Region (Avellino, Benevento, Caserta, Napoli (resulted from the combination of ASL Napoli 1 Centro, Napoli 2 Nord, and Napoli 3 Sud), and Salerno), standardised morbidity ratios (SMR) were calculated, considering the regional population as standard population (indirect standardisation). To calculate 95% confidence intervals (95%CI) for the standardised rates, the Byar’s approximation method based on the Poisson distribution was used. Provincial differences in the MS prevalence were also evaluated with a Poisson regression-based model, with robust estimation of the variance and adjusted for age and sex [31].

Finally, to estimate the proportion of undetected cases (aim 3), we used the capture-recapture method, which employs log-linear models including main effects and specific interaction terms to assess dependence between sources. Capture-recapture has often been used as an indirect method to estimate incidence and prevalence of a condition considering the overlap between more than one data source [32,33,34,35,36]. Model selection was based on a number of parameters including the Akaike Information Criterion (AIC), the Bayesian Information Criterion (BIC) and the goodness-of-fit based confidence intervals following the method published by Regal and Hook [35,36,37].

Statistical analyses were performed using Stata 15.0. Results were considered statistically significant for *p* < 0.05.

### 2.5. Data Availability

Data is available upon request to the Regional Healthcare Society (So.Re.Sa–www.soresa.it).

## 3. Results

The algorithm identifying individuals with at least one MS record during the study period included 5362 MS cases, with 99.0% sensitivity (95%CI = 98.3%, 99.4%), 87.4% specificity (95%CI = 86.4%, 88.5%), 0.93 ROC area (95%CI = 0.92, 0.93), 74.7% positive predictive value (95%CI = 72.7%, 76.6%), 99.6% negative predictive value (95%CI = 99.3%, 99.8%), and 0.78 κ. The algorithm identifying individuals with at least two MS records during the study period captured 4991 MS cases (93% of the sample with one MS record), with 99.1% sensitivity (95%CI = 98.5%, 99.5%), 87.0% specificity (95%CI = 85.9%, 88.1%), 0.93 ROC area (95%CI = 0.92, 0.93), 75.7% positive predictive value (95%CI = 73.7%, 77.6%), 99.6% negative predictive value (95%CI = 99.3%, 99.8%), and 0.78 κ. Considering that both algorithms had a very similar ability to discriminate people with MS from the clinical registry, we favoured the algorithm considering at least one MS record in light of the larger sample identified. 

**Figure 2:**
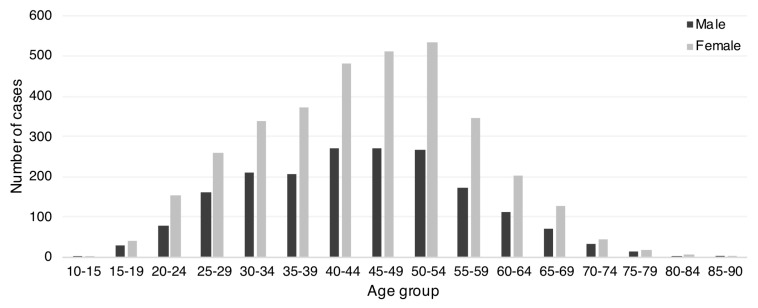
Prevalence of multiple sclerosis (MS) by age and sex.

We identified 5362 people with MS (females = 64.4%; female-to-male ratio = 1.8; age = 44.6 ± 12.9 years). Age and sex distribution are reported in Figure 2. Standardised prevalence rate per 100,000 people was 89.8 cases (95%CI = 87.4, 92.2) (111.8 for females [95%CI = 1 08.1, 115.6] and 66.2 for males [95%CI = 63.2, 69.2]).

The bar graph shows the number of MS individuals living in the Campania Region of Italy on the prevalence day (1 January 2018) by age group and sex.

**Figure 3:**
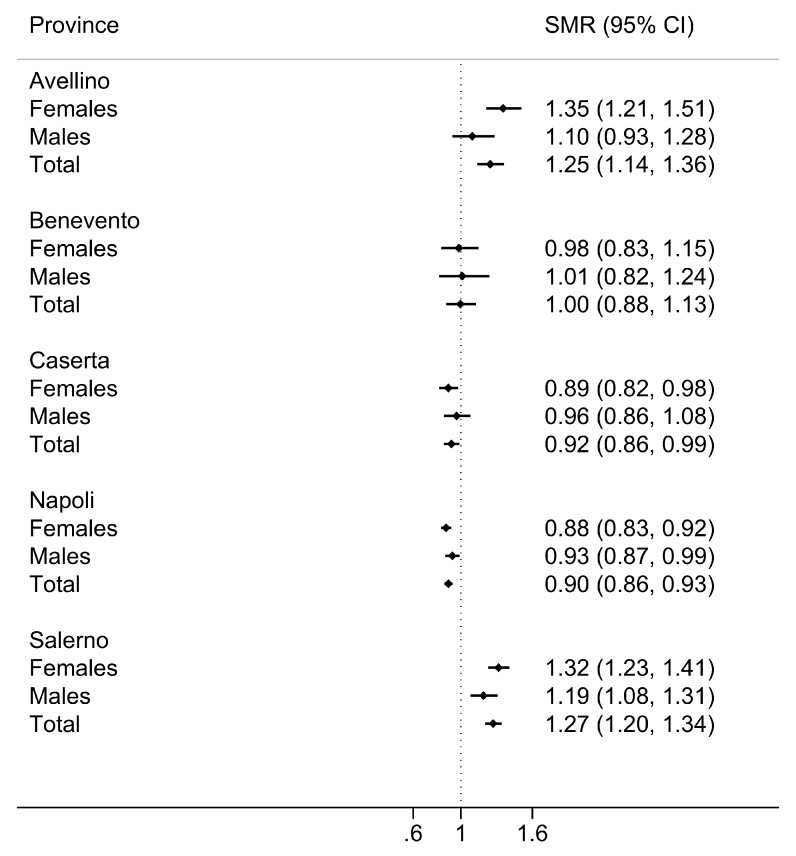
Prevalence of MS in the Provinces of the Campania Region.

The lowest SMR was found in the province of Naples, the most densely populated (SMR = 0.89; 95%CI = 0.86, 0.93), whilst the highest SMR was found in the province of Salerno (SMR = 1.27; 95%CI = 1.20, 1.34). SMRs in the five provinces of the Campania Region are reported in Figure 3. These results were confirmed by regression-based estimation showing that, when compared with the province of Naples, the prevalence was 42% greater in the provinces of Salerno and Avellino (prevalence ratio = 1.42; 95%CI = 1.39, 1.45; and prevalence ratio = 1.42; 95%CI = 1.38, 1.46 respectively), 13% greater in the province of Benevento (prevalence ratio = 1.13; 95%CI = 1.09, 1.18), and 3% greater in the province of Caserta (prevalence ratio = 1.03; 95%CI = 1.01, 1.06).

In the capture-recapture analysis, the model with the best fit showed that the number of expected MS cases was equal to 5508 (95%CI = 5480, 5539), with a 2.7% increase, when compared with the number of detected cases.

The forest plot shows standardised morbidity ratios (SMR) by sex for the five provinces of the Campania Region (Avellino, Benevento, Caserta, Napoli (resulted from the combination of ASL Napoli 1 Centro, Napoli 2 Nord, and Napoli 3 Sud), and Salerno), considering the regional population as the standard population. Ninety-five percent confidence intervals (95%CI) were calculated using the Byar’s approximation method based on the Poisson distribution.

## 4. Discussion

We have validated a case-finding algorithm to capture individuals with MS from routinely collected healthcare data, and, based on this, we estimated the prevalence of MS in the Campania Region (South Italy). After linkage to a clinical registry, our algorithm showed high sensitivity (99.0%), with only 2.7% of MS cases remaining undetected on capture-recapture models [32,33,34,35,36]. The Campania Region accounts for 10% of the Italian population, and thus we captured a large, and possibly fully representative, sample of Italian MS patients.

Previously published MS case-finding algorithms to identify people with MS from administrative healthcare databases used one to three MS records for case identification [26,27,28,29]. Our algorithm used one MS record for case identification and had high sensitivity (99.0%) at detecting MS cases from a clinical registry. In particular, previous studies using similar algorithms presented with the same or frequently lower sensitivity in MS (85.0%–99.0%) [27,38,39], and an in other neurological diseases (e.g., 75.8%–91.2% in Parkinson’s disease, and 85.9%–87.3% in epilepsy) [21]. We have also estimated specificity, positive predictive value, and negative predictive value that, however, should be interpreted cautiously since it specifically relates to the clinical registry rather than to a general individual with/without MS from the Campania Region. Of note, our algorithm was developed considering Italian recommendations for case identification [21], and equally weighted the use of DMTs and other healthcare resources (e.g., hospital admissions, outpatients). Indeed, most algorithms proposed in the last years to identify MS cases from routinely collected healthcare data relied on drug prescriptions (and related consultations to MS Centres), biasing the inclusion towards early MS patients [21]. As such, our algorithm is also applicable to other fields of medicine and is currently under investigation to study other neurological (e.g., epilepsy, headache) and non-neurological diseases (e.g., kidney failure) in the Campania Region. However, the applicability of our algorithm to other Italian Regions should be evaluated carefully, since in the Campania Region all datasets are provided by the Regional Healthcare Society (So.Re.Sa), whilst other Regions rely on different agencies, possibly with heterogeneity in data collection.

Our estimate of 90 MS cases per 100,000 people perfectly falls within prevalence intervals for South European countries, as measured by the Global Burden of Diseases study (60–120 per 100,000) [1]. However, a number of previous Italian studies estimated higher MS prevalence in Italy, being, on average, 176/100,000 in mainland and Sicily, and 299/100,000 in Sardinia [27,38,40,41]. Our lower prevalence estimates are possibly a consequence of different factors. First, the Campania Region is located in South Italy at a low-risk latitude [1], and thus prevalence could be actually lower when compared with other regions from North and Central Italy. Genetic background can also account for differences in prevalence, as in the case of high prevalence in Sardinia [38,42]. There are also methodological differences in our study, as discussed below.

Looking at previous national and international studies using routinely collected healthcare data, our approach holds novelties due to the application of a conservative case-finding algorithm [21], and to the linkage to a clinical dataset, which has been hereby used for algorithm validation and could be used in the future also for clinical validation (e.g., identification of relapses and disability levels). In particular, we included a clinical registry for validation purposes, but not for prevalence estimates as in previous Italian studies [39,40,41,43,44]. In our study, prevalence was measured directly on the population and was not derived from expected annual increase in prevalence adjusted by mortality [38]. Not least, we excluded MS patients who were receiving care in the Campania Region but were actually living elsewhere, which could have overinflated MS prevalence in other Italian studies [14,27]. Other international cohorts included a nationwide cohort in France (>100,000 individuals with MS) [16], and a cohort representative of the UK population (>10,000 individuals with MS) [26], which, however, lack linkage to clinical datasets for validation of case-finding algorithms and clinical outcomes. In Germany, a cohort of >30,000 individuals has been described, though limited by the lack of linkage to clinical datasets and by the risk of multiple counting of patients who changed of insurance number during the study period [45]. In the US, routinely collected healthcare data is derived from insurance claims with an unspecified number of individuals being excluded [46]. As such, our cohort, though smaller than previous international studies, looks representative of the Italian population and holds the potential of higher granularity in both healthcare resource utilisation and clinical perspectives.

Population demographics are in line with previous studies [28,47]. In particular, we found most cases between 40 and 60 years, with 1.8 male-to-female ratio, progressively growing over the life span [1,2]. Of note, we have hereby included age at prevalence date/study inclusion (1-Jan-2018), whilst age at onset would need a specific validation study. We have also found a limited number of paediatric MS cases which would deserve further validation of the algorithm due to possible differences when compared with adults. We favoured the algorithm considering at least one MS record in light of the larger sample identified, when compared with two MS records. On the contrary, to identify paediatric MS with high sensitivity, given the high risk of misclassification (e.g., monophasic demyelinating diseases) [2], the number of MS records should probably increase (e.g., two or more MS records) and the follow-up should be longer.

Limitations of the present study include the possibility of false negatives; though unlikely [16,21,27,47], some patients may have not accessed any MS-related service over three years (e.g., no DMTs, hospital admissions, outpatients with MS exemption), and might have been missed from our case-finding algorithm. In the Italian NHS, there is an overlap between payment exemptions, with the possibility that one person holds more than one reason for exemption; for instance, low household income exemption could be used instead of the MS exemption, thus limiting our case-finding algorithm, especially in high deprivation areas (e.g., the province of Naples and Caserta). We cannot exclude coding errors and data omissions that, however, would have been responsible for wrong compensations and thus would have been very likely detected during the administrative processing. We did not present specific clinical and treatment data, which would need further validation studies (e.g., incident MS, relapse occurrence, etc.) and consistent study objectives (e.g., treatment switch/discontinuation).

## 5. Conclusions

We have validated an algorithm for capturing MS cases from routinely collected healthcare data in the Campania Region (South Italy) and have confirmed expected rates of prevalence. In the future, this cohort will allow studies with high granularity of clinical, environmental, healthcare resource utilisation, and pharmacoeconomic variables on a large sample, representing 10% of the Italian population. Not least, the possibility of data linkage to a clinical dataset will give the opportunity of integrating routinely collected healthcare data with clinical variables and patient-reported outcome measures.

## References

[1] Wallin M.T., Culpepper W.J., Nichols E., Bhutta Z.A., Gebrehiwot T.T., Hay S.I., Khalil I.A., Krohn K.J., Liang X., Naghavi M. (2019). Global, regional, and national burden of multiple sclerosis 1990–2016: A systematic analysis for the Global Burden of Disease Study 2016. Lancet Neurol..

[2] Thompson A.J., Baranzini S.E., Geurts J., Hemmer B., Ciccarelli O. (2018). Multiple sclerosis. Lancet.

[3] De Angelis F., John N., Brownlee W. (2018). Disease-modifying therapies for multiple sclerosis. BMJ.

[4] Tur C., Moccia M., Barkhof F., Chataway J., Sastre-Garriga J., Thompson A.J., Ciccarelli O. (2018). Assessing treatment outcomes in multiple sclerosis trials and in the clinical setting. Nat. Rev. Neurol..

[5] Thalheim C. (2015). Pooling real-world multiple sclerosis patient data on a European level: A true story of success. Neurodegener. Dis. Manag..

[6] Glaser A., Stahmann A., Meissner T., Flachenecker P., Horáková D., Zaratin P., Brichetto G., Pugliatti M., Rienhoff O., Vukusic S. (2019). Multiple Sclerosis Registries in Europe—An Updated Mapping Survey. Mult. Scler. Relat. Disord..

[7] Trojano M., Bergamaschi R., Amato M.P., Comi G., Ghezzi A., Lepore V., Marrosu M.G., Mosconi P., Patti F., Ponzio M. (2019). The Italian multiple sclerosis register. Neurol. Sci..

[8] Moccia M., Tajani A., Acampora R., Signoriello E., Corbisiero G., Vercellone A., Sergianni P., Pennino F., Lanzillo R., Palladino R. (2019). Healthcare resource utilization and costs for multiple sclerosis management in the Campania Region of Italy: Comparison between centre-based and local service healthcare delivery. PLoS ONE.

[9] Sorensen P.S., Giovannoni G., Montalban X., Thalheim C., Zaratin P., Comi G. (2019). The Multiple Sclerosis Care Unit. Mult. Scler. J..

[10] Kobelt G., Thompson A., Berg J., Gannedahl M., Eriksson J. (2017). New insights into the burden and costs of multiple sclerosis in Europe. Mult. Scler. J..

[11] Trojano M., Tintore M., Montalban X., Hillert J., Kalincik T., Iaffaldano P., Spelman T., Sormani M., Butzkueven H. (2017). Treatment decisions in multiple sclerosis - insights from real-world observational studies. Nat. Rev. Neurol..

[12] Kalincik T., Butzkueven H. (2016). Observational data: Understanding the real MS world. Mult. Scler. J..

[13] Cohen J.A., Trojano M., Mowry E.M., Uitdehaag B.M.J., Reingold S.C., Marrie R.A. (2020). Leveraging real-world data to investigate multiple sclerosis disease behavior, prognosis, and treatment. Mult. Scler. J..

[14] Bezzini D., Ulivelli M., Gualdani E., Razzanelli M., Ferretti F., Meucci G., Francesconi P., Battaglia M.A. (2020). Increasing prevalence of multiple sclerosis in Tuscany, Italy. Neurol. Sci..

[15] Birnbaum H.G., Cremieux P.Y., Greenberg P.E., LeLorier J., Ostrander J.A., Venditti L. (1999). Using healthcare claims data for outcomes research and pharmacoeconomic analyses. Pharmacoeconomics.

[16] Roux J., Guilleux A., Lefort M., Leray E. (2019). Use of healthcare services by patients with multiple sclerosis in France over 2010–2015: A nationwide population-based study using health administrative data. Mult. Scler. J. Exp. Transl. Clin..

[17] Fox E., Vieira M.C., Johnson K., Peeples M., Bensimon A.G., Signorovitch J., Herrera V. (2019). Real-world durability of relapse rate reduction in patients with multiple sclerosis receiving fingolimod for up to 3 years: A retrospective US claims database analysis. J. Neurol. Sci..

[18] Signorelli C., Odone A., Oradini-Alacreu A., Pelissero G. (2019). Universal Health Coverage in Italy: Lights and shades of the Italian National Health Service which celebrated its 40th anniversary. Health Policy.

[19] Agenzia Italiana del Farmaco (AIFA) Banca Dati Farmaci. https://www.farmaci.agenziafarmaco.gov.it/.

[20] European Medicine Agency (EMA) European Public Assessment Reports. https://www.ema.europa.eu/en/medicines.

[21] Canova C., Danieli S., Amidei C.B., Simonato L., Di Domenicantonio R., Cappai G., Bargagli A.M. (2019). A systematic review of case-identification algorithms based on Italian healthcare administrative databases for three relevant diseases of the nervous system: Parkinson’s disease, multiple sclerosis, and epilepsy. Epidemiol. Prev..

[22] Moccia M., Lanzillo R., Palladino R., Chang K.K.C.M.K.C.-M., Costabile T., Russo C., De Rosa A., Carotenuto A., Saccà F., Maniscalco G.T.G.T. (2016). Cognitive impairment at diagnosis predicts 10-year multiple sclerosis progression. Mult. Scler. J..

[23] Moccia M., Capacchione A., Lanzillo R., Carbone F., Micillo T., Perna F., De Rosa A., Carotenuto A., Albero R., Matarese G. (2019). Coenzyme Q10 supplementation reduces peripheral oxidative stress and inflammation in Interferon-Beta1a treated multiple sclerosis. Ther. Adv. Neurol. Disord..

[24] Claflin S.B., Broadley S., Taylor B.V. (2019). The effect of disease modifying therapies on disability progression in multiple sclerosis: A systematic overview of meta-analyses. Front. Neurol..

[25] Moccia M., Palladino R., Carotenuto A., Saccà F., Russo C.V., Lanzillo R., Brescia Morra V. (2018). A 8-year retrospective cohort study comparing Interferon-β formulations for relapsing-remitting multiple sclerosis. Mult. Scler. Relat. Disord..

[26] Palladino R., Marrie R., Majeed A., Chataway J. (2020). Evaluating the Risk of Macrovascular Events and Mortality Among People With Multiple Sclerosis in England. JAMA Neurol..

[27] Bezzini D., Policardo L., Meucci G., Ulivelli M., Bartalini S., Profili F., Battaglia M.A., Francesconi P. (2016). Prevalence of multiple sclerosis in tuscany (central Italy): A study based on validated administrative data. Neuroepidemiology.

[28] Culpepper W.J., Marrie R.A., Langer-Gould A., Wallin M.T., Campbell J.D., Nelson L.M., Kaye W.E., Wagner L., Tremlett H., Chen L.H. (2019). Validation of an algorithm for identifying MS cases in administrative health claims datasets. Neurology.

[29] Disanto G., Zecca C., MacLachlan S., Sacco R., Handunnetthi L., Meier U.C., Simpson A., McDonald L., Rossi A., Benkert P. (2018). Prodromal symptoms of multiple sclerosis in primary care. Ann. Neurol..

[30] Sim J., Wright C.C. (2005). The Kappa Statistic in Reliability Studies: Use, Interpretation, and Sample Size Requirements. Phys. Ther..

[31] Barros A.J.D., Hirakata V.N. (2003). Alternatives for logistic regression in cross-sectional studies: An empirical comparison of models that directly estimate the prevalence ratio. BMC Med. Res. Methodol..

[32] El Adssi H., Debouverie M., Guillemin F. (2012). Estimating the prevalence and incidence of multiple sclerosis in the Lorraine region, France, by the capture-recapture method. Mult. Scler. J..

[33] Farcomeni A. (2020). Population size estimation with interval censored counts and external information: Prevalence of multiple sclerosis in Rome. Biom. J..

[34] International Working Group for Disease Monitoring and Forecasting (1995). Capture-recapture and Multiple-Record Systems Estimation I: History and Theoretical Development. Am. J. Epidemiol..

[35] International Working Group for Disease Monitoring and Forecasting (1995). Capture-recapture and multiple-record systems estimation II: Applications in human diseases. Am. J. Epidemiol..

[36] Hook E., Regal R. (1995). Capture-recapture Methods in Epidemiology: Methods and Limitations. Epidemiol. Rev..

[37] Regal R.R., Hook E.B. (1984). Goodness-of-fit based confidence intervals for estimates of the size of a closed population. Stat. Med..

[38] Battaglia M.A., Bezzini D. (2017). Estimated prevalence of multiple sclerosis in Italy in 2015. Neurol. Sci..

[39] Bargagli A.M., Colais P., Agabiti N., Mayer F., Buttari F., Centonze D., Di Folco M., Filippini G., Francia A., Galgani S. (2016). Prevalence of multiple sclerosis in the Lazio region, Italy: Use of an algorithm based on health information systems. J. Neurol..

[40] Grassivaro F., Puthenparampil M., Pengo M., Saiani M., Venturini M., Stropparo E., Perini P., Rinaldi F., Freddi N., Cadaldini M. (2019). Multiple sclerosis incidence and prevalence trends in the Province of Padua, Northeast Italy, 1965-2018. Neuroepidemiology.

[41] Solaro C., Ponzio M., Moran E., Tanganelli P., Pizio R., Ribizzi G., Venturi S., Mancardi G.L., Battaglia M.A. (2015). The changing face of multiple sclerosis: Prevalence and incidence in an aging population. Mult. Scler. J..

[42] Steri M., Orrù V., Idda M.L., Pitzalis M., Pala M., Zara I., Sidore C., Faà V., Floris M., Deiana M. (2017). Overexpression of the Cytokine BAFF and Autoimmunity Risk. N. Engl. J. Med..

[43] Nicoletti A., Rascunà C., Boumediene F., Vasta R., Cicero C.E., Lo Fermo S., Ferrante M., Marziolo R., Maimone D., Grimaldi L.M. (2020). Incidence of multiple sclerosis in the province of Catania. A geo-epidemiological study. Environ. Res..

[44] Bergamaschi R., Monti M.C., Trivelli L., Introcaso V.P., Mallucci G., Borrelli P., Gerosa L., Montomoli C. (2020). Increased prevalence of multiple sclerosis and clusters of different disease risk in Northern Italy. Neurol. Sci..

[45] Höer A., Schiffhorst G., Zimmermann A., Fischaleck J., Gehrmann L., Ahrens H., Carl G., Sigel K., Osowski U., Klein M. (2014). Multiple sclerosis in Germany: Data analysis of administrative prevalence and healthcare delivery in the statutory health system. BMC Health Serv. Res..

[46] Dilokthornsakul P., Valuck R.J., Nair K.V., Corboy J.R., Allen R.R., Campbell J.D. (2016). Multiple sclerosis prevalence in the United States commercially insured population. Neurology.

[47] Wallin M.T., Culpepper W.J., Campbell J.D., Nelson L.M., Langer-Gould A., Marrie R.A., Cutter G.R., Kaye W.E., Wagner L., Tremlett H. (2019). The prevalence of MS in the United States: A population-based estimate using health claims data. Neurology.

